# Tactile temporal predictions: The influence of conditional probability

**DOI:** 10.1177/20416695241264736

**Published:** 2024-07-23

**Authors:** Rongxia Ren, Weichao An, Yinghua Yu, Xiaoyu Tang, Yoshimichi Ejima, Jinglong Wu, Jiajia Yang

**Affiliations:** Graduate School of Interdisciplinary Science and Engineering in Health Systems, 12997 Okayama University, Okayama, Japan; Graduate School of Interdisciplinary Science and Engineering in Health Systems, 12997 Okayama University, Okayama, Japan; Graduate School of Interdisciplinary Science and Engineering in Health Systems, 12997 Okayama University, Okayama, Japan; School of Psychology, Liaoning Collaborative Innovation Center of Children and Adolescents Healthy Personality Assessment and Cultivation, 66523 Liaoning Normal University, Dalian, China; Graduate School of Interdisciplinary Science and Engineering in Health Systems, 12997 Okayama University, Okayama, Japan; Graduate School of Interdisciplinary Science and Engineering in Health Systems, 12997 Okayama University, Okayama, Japan; Graduate School of Interdisciplinary Science and Engineering in Health Systems, 12997 Okayama University, Okayama, Japan

**Keywords:** temporal prediction, foreperiod, conditional probability, touch

## Abstract

Predicting the timing of incoming information allows brain to optimize information processing in dynamic environments. However, the effects of temporal predictions on tactile perception are not well established. In this study, two experiments were conducted to determine how temporal predictions interact with conditional probabilities in tactile perceptual processing. In Experiment 1, we explored the range of the interval between preceding ready cues and imperative targets in which temporal prediction effects can be observed. This prediction effect was observed for intervals of 500 and 1,000 ms. In Experiment 2, we investigated the benefits of temporal predictions on tactile perception while manipulating the conditional probability (setting the stimulus onset earlier or later than the predicted moment in short and long intervals). Our results revealed that this effect became stronger as the probability of the stimulus at the predicted time point increased under short-interval conditions. Together, our results show that the difficulty of transferring processing resources increases in temporally dynamic environments, suggesting a greater subjective cost associated with maladaptive responses to temporally uncertain events.

In complex and dynamic environments, perceiving can be guided flexibly by predicting when relevant information will appear (i.e., temporal prediction). The ability to perform and update actions according to temporal predictions is essential for enhanced behavioral performance, that is, faster reaction times (RTs) and higher accuracy for expected targets (Amit et al., 2019; Bolger et al., 2014; Nobre & Van Ede, 2018; Oswal et al., 2007; Sanabria & Correa, 2013), and neuronal activity (Herbst & Obleser, 2019; Rohenkohl & Nobre, 2011; Rolke et al., 2016; Yu et al., 2019, 2022). Previous studies on this topic have mainly been conducted in visual and auditory domains. In comparison, the effects of temporal prediction on tactile perception are not well established, and little is known about how expecting a stimulus at a specific time can enhance its perceptual processing.

Temporal predictions could be induced by rhythmic stimulation (Abeles et al., 2020; Barne et al., 2022; Bouwer et al., 2019; Jaramillo & Zador, 2011; Jones, 2019), as the onset times of each stimulus in a rhythmic sequence are highly predictable (Wilsch et al., 2015). This form of stimulus presentation is often referred to as an isochronous rhythm, and temporal predictions are generated based on the periodic presentation of stimuli (Rimmele et al., 2018). The foreperiod paradigm is one of the most commonly used designs in temporal prediction research (Korolczuk et al., 2024; Pfeuffer et al., 2020; Shdeour et al., 2024). The foreperiod refers to the interval between preceding ready cues and imperative targets (Los et al., 2001). In the present study, tactile temporal predictions were generated by presenting rhythmic stimuli in the foreperiod paradigm. During rhythmic blocks, the foreperiod duration remains constant across trials (predicted condition), allowing for temporal prediction of target onset. In contrast, the foreperiod can vary within an arrhythmic block (unpredicted condition), in which the temporal occurrence of the imperative stimulus is not predictable. That is, in the variable foreperiod paradigm, the target could appear either at the predicted moment or earlier or later than the predicted moment, which leads to different probability distributions in various foreperiods.

How do different probability distributions across various foreperiods influence tactile temporal prediction? Although brain has been shown to encode the probability of a target appearing (Basso & Wurtz, 1997), little is known about how such probabilities are represented by brain when they change as a function of time (Janssen & Shadlen, 2005). As time passes, the probability of an event occurring at a variable foreperiod could change. If no event has occurred at a certain given time, the probability of occurrence during subsequent longer foreperiods would increase. This means that predictions for the event's onset would improve over time (Coull et al., 2016; Cravo et al., 2011; Duyar et al., 2024; Tal-Perry & Yuval-Greenberg, 2022). Based on this, the responses would be faster when the target was present later than at the predicted moment.

The present study investigated the benefits of temporal prediction on tactile perception while manipulating the probability of a stimulus appearing at a given moment. First, we investigated how temporally precise such temporal predictions are and in which range of foreperiods such effects can be found. In Experiment 1, temporal predictions were manipulated by randomly setting a constant or variable foreperiod duration in each trial from the four possible foreperiods (i.e., 500, 1,000, 1,500, and 2,000 ms). We expected faster and more accurate detection of targets at predicted moments compared to unpredicted moments for all four possible foreperiods. Additionally, the temporal prediction effect may be weakened with increasing foreperiod duration. Second, we aimed to determine whether and how temporal predictions in specific foreperiods are influenced by the stimulus probability. In Experiment 2, warning cues and targets were repeatedly presented at specific foreperiods to establish temporal predictions. By utilizing this rhythmic sequence, we guide predictions to specific points in time. Moreover, the target may be presented earlier or later than the specific time point. This approach is beneficial for evaluating the temporal prediction effect. We also manipulated the probability of the target appearing at a specific time point (i.e., 75%, 50%, and 25%). We expected faster and more accurate target detection at specific time points for different probabilities. Additionally, the temporal prediction effect may be enhanced with increasing probability.

## Experiment 1

### Materials and Methods

#### Participants

Sixteen participants (4 females and 12 males) between 22 and 32 years of age (average age = 25.5 years, *SD* = 2.90) participated in the experiment. All participants reported normal hearing, and had no tactile or motor impairments. The experimental protocol was approved by the local Medical Ethics Committee at Okayama University Hospital (ethics number: 1702-030). The experiment was conducted in accordance with the relevant guidelines and regulations, and all participants signed informed consent documents prior to the experiment.

#### Apparatus and Stimuli

**Figure 1.**
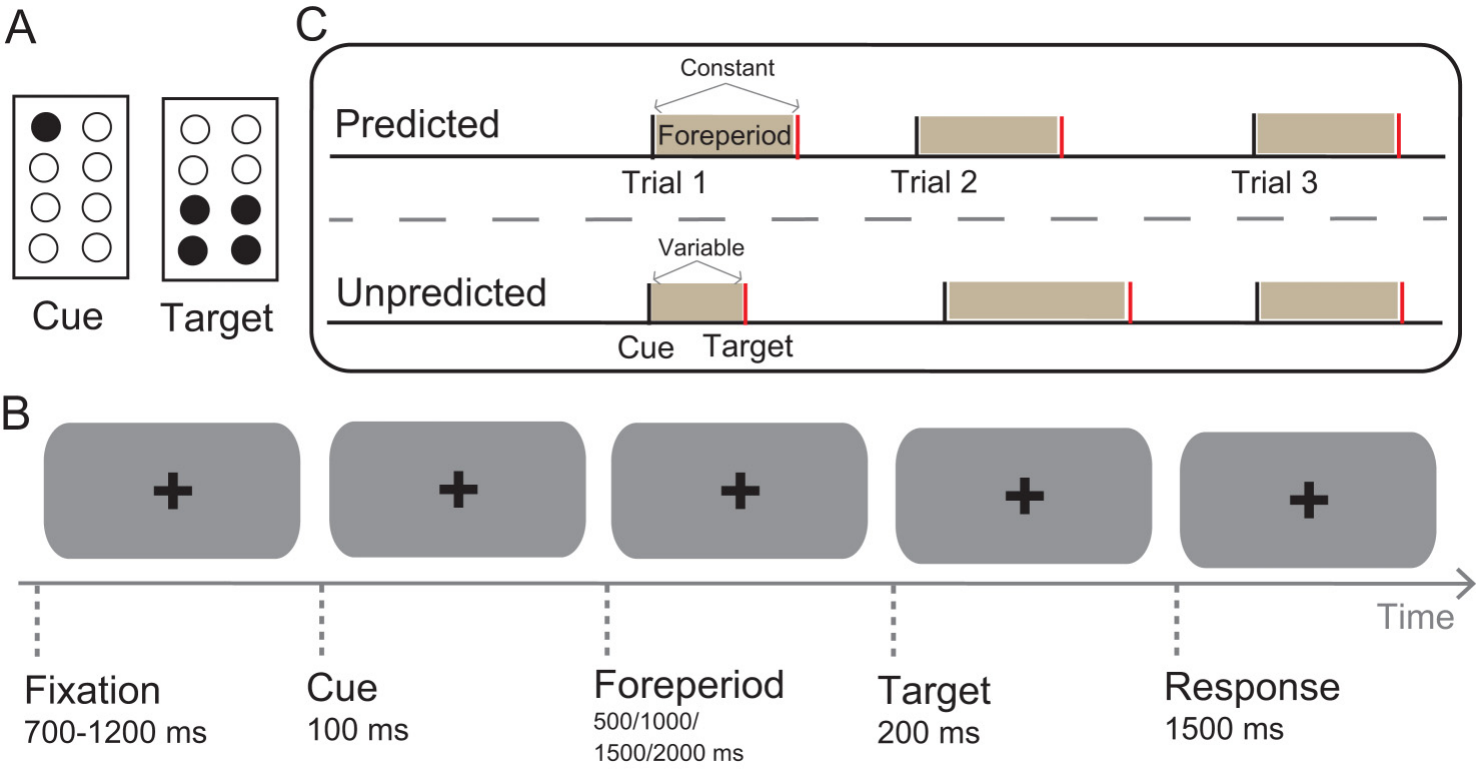
Illustration of the experimental stimuli and procedure. (A) Schematic of the tactile stimulus patterns applied to the 1st segment of the index finger. (B) Experimental procedure. (C) The intervals between the cue and target (foreperiods) were constant in the predicted condition and varied among four options in the predicted condition.

*Note.* The black dots in (A) indicate that the pins had risen.

The participants sat at a table and rested their hands on the table surface. Their head was supported by a chin and forehead rest, and their eyes were covered by blindfolds. Tactile stimuli were applied to the distal phalanx of the right index finger using a Braille piezostimulator (SC9 equipment, KGS Company, Japan). The stimulator had eight individually controllable plastic pins, which were grouped in a 2 × 4 array (Figure 1A). Using a custom-built electrical drive, pins could be elevated from the resting position by 0.7 mm. In the present experiment, the tactile stimulation consisted of a cue stimulus and a target stimulus (Figure 1A). For the cue stimulus, a pin in the first row was elevated, and for the target stimulus, four pins in the last two rows were elevated. The cue and target stimuli were always applied to the same finger. A black fixation cross was centrally displayed on a gray background on a monitor at a distance of 60 cm from the participant. Participants wore headphones (Wh-1000xm4, Sony, Japan) to reduce the influence of sounds produced by the tactile stimulators. The experimental program was written in MATLAB (MathWorks, Natick, MA, USA), and the Psychtoolbox was used.

#### Procedure and Design

The experimental procedure is illustrated in Figure 1B. Each trial began with a random interval of 700–1,200 ms, ensuring that the sequence of tactile stimuli was nonrhythmic across trials (Badde et al., 2020). Then, the tactile cue was presented for 100 ms, marking the onset of the foreperiod (500/1,000/1,500/2,000 ms). After the foreperiod, either the tactile target was presented for 200 ms or no stimulus was presented. The participants were instructed to respond to the tactile target stimulus by pressing a response button as quickly and accurately as possible with the index finger of their left hand. The response duration was limited to 1,500 ms, and the next trial started immediately after the response had been registered.

Two factors, the foreperiod duration and stimulus predictability, were manipulated in the experiment. The trials varied with respect to the length of the foreperiod between the cue and target (500, 1,000, 1,500, and 2,000 ms). The foreperiod was either constant (predicted condition) or variable (unpredicted condition) across trials within a block. These two conditions were varied between blocks (Figure 1C). Following one practice block, each participant completed eight experimental blocks in a random order for a total of 800 trials. The catch trials (no target appearance) comprised approximately 10% of the total trials and were included in the experimental procedure. Thus, there were 90 trials for each experimental condition. Each of the four foreperiods was presented 90 times, with either all repetitions of one foreperiod in the same block (predicted condition) or all foreperiods presented at equal rates (uniform probability) in a random order within blocks (unpredicted condition). The participants were allowed to take a 5-min break between blocks. The total time for the experiment was approximately 50 min.

#### Data Analysis

Regarding data trimming, the practice trials and catch trials were not considered in the analyses. In addition, trials with no responses were excluded. Trials with RTs exceeding the mean  ± 3 *SDs* (3 *SDs* above/below the mean per participant and condition) were excluded. A total of 5.95% of the original data were removed after data trimming.

A 4 (foreperiods: 500, 1,000, 1,500, and 2,000 ms) × 2 (predictability: predicted and unpredicted) repeated-measures analysis of variance (ANOVA) was conducted for hit rate, false alarm rate, and RTs using the R package in RStudio (RStudio Team, 2021). The Greenhouse–Geisser epsilon correction was used to correct for nonsphericity. The Bonferroni correction was applied for post-hoc comparisons. The effect size was reported as the partial eta-squared (η_p_^2^) for the ANOVA. One-way repeated-measures ANOVA was used to compare the tactile prediction effect (predicted vs. unpredicted) of the foreperiods (500, 1,000, 1,500, and 2,000 ms).

### Results

#### Hit Rate

Table 1 shows the mean hit rate in each condition. No significant effect of hit rate was observed according to the ANOVA. There were no main effects of predictability [*F*(1, 15) = 2.34, *p* = .147, η_p_^2^ = 0.13] or foreperiod [*F*(1.74, 26.10) = 0.34, *p* = .684, η_p_^2^ = 0.02], and no interaction effect of predictability × foreperiod [*F*(1.94, 29.14) = 1.79, *p* = .185, η_p_^2^ = 0.11].

**Table 1.** The average and standard deviations (in parentheses) of hit rate and false alarm rate (%) for all conditions of prediction and foreperiod.

	Prediction	Foreperiod (ms)			
		500	1,000	1,500	2,000
Hit rate (%)	Predicted	98 (2)	97 (6)	96 (7)	98 (5)
	Unpredicted	95 (7)	97 (6)	97 (6)	96 (6)
False alarm rate (%)	Predicted	20 (18)	11 (20)	7 (14)	2 (4)
	Unpredicted	1 (3)	1 (3)	1 (3)	0 (0)

#### False Alarm Rate

Table 1 shows the mean false alarm rate in each condition. There were the significant main effects of predictability [*F*(1, 15) = 12.74, *p* = .003, η_p_^2^ = 0.46] and foreperiod [*F*(2.57, 38.59) = 6.95, *p* = .001, η_p_^2^ = 0.32], and interaction effect of predictability × foreperiod [*F*(2.33, 35.01) = 5.37, *p* = .007, η_p_^2^ = 0.26]. Pairwise post-hoc comparisons for this interaction revealed that false alarm rates were higher in the predicted (*M* = 20%, *SE* = 4.7%) than in the unpredicted condition for the foreperiod of 500 ms [*M* = 1.2%, *SE* = 0.9%, *t*_(15)_ = 4.20, *p* < .001, *d* = 1.35]. However, the false alarm rate did not differ between the predicted and unpredicted conditions for foreperiods of 1,000, 1,500, or 2,000 ms [1,000 ms: *M*(predicted) = 11.25%, *SE* = 5%, *M*(unpredicted) = 1.25%, *SE* = 0.9%, *t*_(15)_ = 2.03, *p* = .06, *d* = 0.72; 1,500 ms: *M*(predicted) = 7.5%, *SE* = 3.5%, *M*(unpredicted) = 0.63%, *SE* = 0.6%, *t*_(15)_ = 2.11, *p* = .052, *d* = 0.50; 2,000 ms: *M*(predicted) = 1.88%, *SE* = 1%, *M*(unpredicted) = 0, *SE* = 0, *t*_(15)_ = 1.86, *p* = .083, *d* = 0.14].

#### Reaction Times

**Figure 2.**
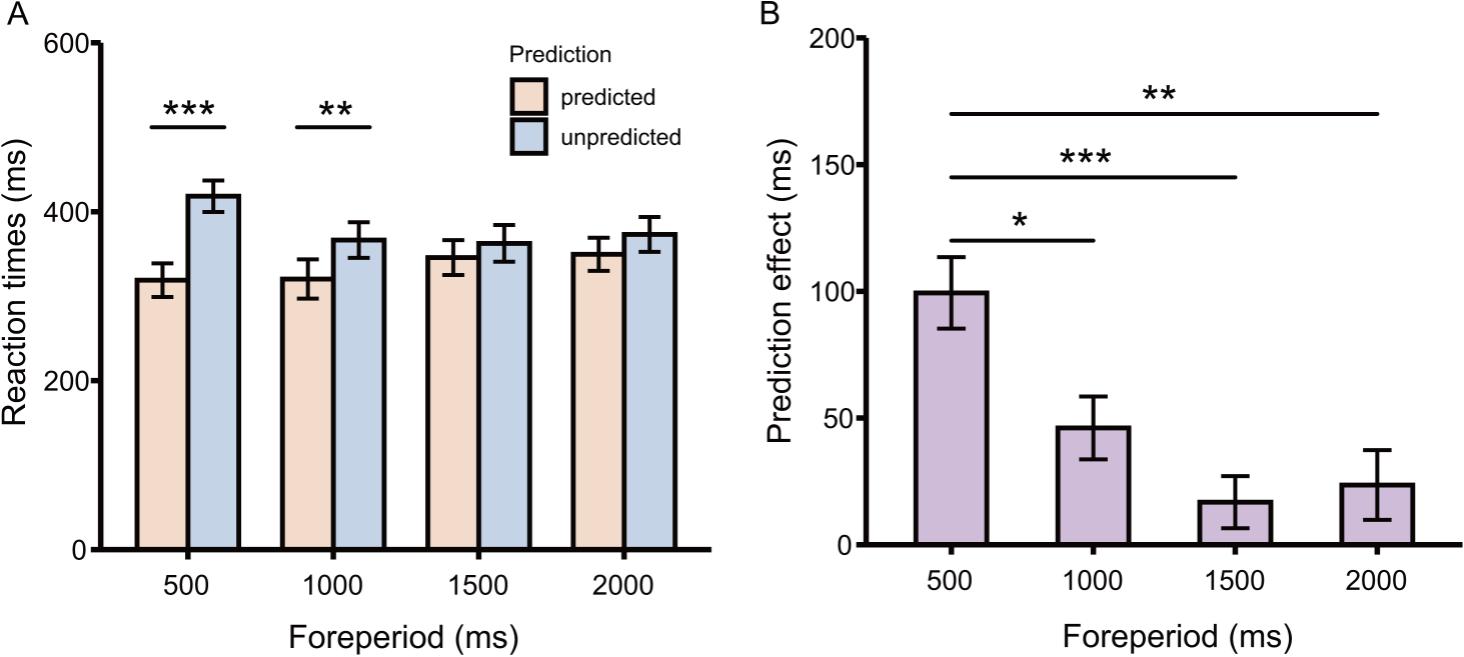
Reaction time results in Experiment 1. (A) Reaction times for unpredicted (light blue) and predicted (light yellow) targets for foreperiods of 500, 1,000, 1,500, and 2,000 ms. (B) Magnitude of prediction effect. Error bars represent standard errors of the means.

*Note.* *p < .05, **p < .01, ***p < .001.

Figure 2A shows the mean RTs in each condition. There was a main effect of predictability [*F*(1, 15) = 26.37, *p* < .001, η_p_^2^ = 0.64], with slower RTs for the predicted trials (*M* = 333.61 ms, *SE* = 19.07) than for the unpredicted trials (*M* = 380.08 ms, *SE* = 19.99), indicating a prediction effect. And there was also a main effect of foreperiod [*F*(2.55, 38.19) = 3.16, *p* = .042, η_p_^2^ = 0.17], with slower RTs for the 500 ms foreperiod trials (*M* = 368.61 ms, *SE* = 17.91) than for the 1,000 ms foreperiod trials (*M* = 343.37 ms, *SE* = 21.32, *t*_(15)_ = 3.44, *p* = .022, *d* = 0.51).

Importantly, there was a predictability × foreperiod interaction effect [*F*(2.33, 34.96) = 13.13, *p* < .001, η_p_^2^ = 0.47]. Pairwise comparisons for this interaction revealed that RTs were shorter in the predicted condition than in the unpredicted condition for foreperiods of 500 and 1,000 ms [500 ms: *M*(predicted) = 318.91 ms, *SE* = 19.88, *M*(unpredicted) = 418.30 ms, *SE* = 18.60, *t*_(15)_ = 7.04, *p* < .001, *d* = 1.99; 1,000 ms: *M*(predicted) = 320.33 ms, *SE* = 23.25, *M*(unpredicted) = 366.42 ms, *SE* = 21.11, *t*_(15)_ = 3.72, *p* = .002, *d* = 0.93]. However, the RTs did not differ between the predicted and unpredicted conditions for foreperiods of 1,500 or 2,000 ms [1,500 ms: *M*(predicted) = 345.70 ms, *SE* = 20.62, *M*(unpredicted) = 362.49 ms, *SE* = 21.74, *t*_(15)_ = 1.64, *p* = .12, *d* = 0.34; 2,000 ms: *M*(predicted) = 349.52 ms, *SE* = 19.61, *M*(unpredicted) = 373.09 ms, *SE* = 20.60, *t*_(15)_ = 1.72, *p* = .11, *d* = 0.47].

A one-way (foreperiods: 500, 1,000, 1,500, and 2,000 ms) repeated-measures ANOVA was conducted for prediction effect (Figure 2B). A main effect of foreperiods [*F*(2.33, 34.96) = 13.13, *p* < .001, η_p_^2^ = 0.47] was found, with greater prediction effect for 500 ms (*M* = 99.38 ms, *SE* = 14.12) compared to 1,000 ms (*M* = 46.10 ms, *SE* = 12.41, *t*_(15)_ = 3.63, *p* = .015, *d* = 0.91), 1,500 ms (*M* = 16.80 ms, *SE* = 10.27, *t*_(15)_ = 5.33, *p* < .001, *d* = 1.41), and 2,000 ms (*M* = 23.57 ms, *SE* = 13.75, *t*_(15)_ = 3.99, *p* = .007, *d* = 1.30).

In summary, tactile temporal prediction can be formed with the foreperiod paradigm with inter-foreperiod durations as short as 500 ms. The results of false alarm rate indicated that participants were more inclined to make a response in predicted conditions in 500 ms foreperiod. Moreover, this temporal prediction effect occurred with relatively short foreperiods (500 and 1,000 ms). And the magnitude of the prediction effect gradually weakened as the foreperiod duration became longer. Therefore, in Experiment 2, we chose the two time points of 500 and 1,000 ms as short and long intervals, respectively.

## Experiment 2

### Materials and Methods

#### Participants

In this experiment, a power analysis using G*Power (Faul et al., 2009) was established based on effect sizes from Experiment 1 (parameters: η_p_^2^ = 0.17, effect size *f* = 0.45, α = 0.05, 1 − β = 0.9). A minimum of five participants is required. Ultimately, 28 participants (7 females; age range: 21–33 years; mean age: 23.9 ± 2.6 years) were recruited as paid volunteers. All participants were right-handed, had normal hearing, and lacked tactile and motor impairments. The experimental protocol was approved by the local Medical Ethics Committee at Okayama University Hospital (Ethics number: 1702-030). The experiment was conducted in accordance with the relevant guidelines and regulations, and all participants signed informed consent documents prior to the experiment.

#### Task and Design

The apparatus and stimuli employed were identical to those used in Experiment 1. The participants were instructed to respond to the tactile target by pressing a response button as quickly and accurately as possible with the index finger of their left hand. In addition, the procedure was the same as that used in Experiment 1.

However, the experimental design was different from that of Experiment 1. Three factors (predictability, interval, and probability) were manipulated in Experiment 2. The participants completed short-interval and long-interval sessions. In the short-interval session, the predicted foreperiod between the tactile cue and target was 500 ms, and the unpredicted foreperiod was 200 ms (early, i.e., earlier than the predicted moment) or 800 ms (late, i.e., later than the predicted moment). In the long-interval session, the predicted foreperiod between the tactile cue and target was 1,000 ms, and the unpredictable foreperiod was 700 ms (early, i.e., earlier than the predicted moment) or 1,300 ms (late, i.e., later than the predicted moment).

A total of six blocks (120 trials per block) were presented, equally distributed in short/long interval. Each interval condition consisted of three blocks, representing 75%, 50%, and 25% predictability, respectively. Taking short interval as an example, the target was followed by a foreperiod of 200/500/800 ms (early/predicted/late) depending on the experimental condition. In the 75% block, the probability that the target appeared at the predicted interval (500 ms) was set to 75% (90 trials). In the remaining trials, the target appeared at unpredictable intervals of 200 ms (early) or 800 ms (late). In the 50% block, the probability that the target appeared at the predicted interval was set to 50% (60 trials). And, in the 25% block, the probability that the target appeared at the predicted interval (500 ms) was set to 25% (30 trials). The distribution of blocks with long-interval condition was the same as that with short-interval condition. It should be noted that the foreperiod between the tactile cue and target was 700/1,000/1,300 ms (early/predicted/late) in long-interval condition. All participants were balanced between intervals and probabilities, as shown in Table 2.

**Table 2.** Experimental sequence of the participants in Experiment 2.

Sequence	Interval		Block
	First	Second	
1	Short interval	Long interval	25% → 50% → 75% blocks
2	Short interval	Long interval	75% → 50% → 25% blocks
3	Long interval	Short interval	25% → 50% → 75% blocks
4	Long interval	Short interval	75% → 50% → 25% blocks

#### Data Analysis

The data trimming procedure was the same as that in Experiment 1. A participant was removed because the response was too fast, and no RT was recorded. The practice trials were excluded from the analyses. Next, trials with no responses were excluded. Then, trials with RTs exceeding the mean ± 3 *SDs* (3 *SDs* above/below the mean per participant and condition) were excluded. A total of 4.71% of the original data were excluded after data trimming.

A 2 (standard interval: short and long) × 3 (probability: 75%, 50%, and 25%) × 3 (predictability: early, predicted, and late) repeated-measures ANOVA was conducted for the accuracy and RT using the bruceR package in RStudio (RStudio Team, 2021). In a subsequent analysis, the data were reexamined considering the prediction effect, which was calculated by subtracting the mean predicted RT from each mean early (unpredicted) RT to determine the early-prediction effect. Similarly, the late-prediction effect was obtained by subtracting the mean predicted RT from each mean late (unpredicted) RT. Then, to evaluate the prediction effect, a repeated-measures ANOVA with a 2 (standard interval: short and long) × 3 (probability: 75%, 50%, and 25%) × 2 (prediction effect: early-prediction effect and late-prediction effect) within-participant design was used. The Greenhouse–Geisser epsilon correction was used to correct for nonsphericity. The Bonferroni correction was applied for post-hoc comparisons. The effect size was reported as the partial eta-squared (η_p_^2^) for the ANOVA. A 2 (standard interval: short and long) × 3 (probability: 75%, 50%, and 25%) × 2 (prediction effect: early and late prediction) repeated-measures ANOVA was used to evaluate the tactile prediction effect. Early-prediction effect was obtained by subtracting the RT in the early unpredicted condition from that in the predicted condition, that is, early minus predicted moment condition. Late-prediction effect was obtained by subtracting the RT in the late unpredicted condition from that in the predicted condition, that is, late minus predicted moment condition.

### Results

#### Accuracy

Table 3 shows the accuracy versus the predictability in the short- and long-standard intervals for the different probabilities. No significant effect of accuracy was observed according to the ANOVA. There were no main effects of the standard interval [*F*(1, 26) = 0.40, *p* = .535, η_p_^2^ = 0.01], predictability [*F*(1.56, 40.61) = 2.50, *p* = .11, η_p_^2^ = 0.09], or probability [*F*(1.95, 50.74) = 2.10, *p* = .134, η_p_^2^ = 0.07]. Furthermore, there were no interaction effects of the standard interval × predictability × probability [*F*(2.87, 74.59) = 0.024, *p* = .86, η_p_^2^ = 0.01], standard interval × predictability [*F*(1.41, 36.75) = 0.87, *p* = .39, η_p_^2^ = 0.03], standard interval × probability [*F*(1.97, 51.15) = 0.14, *p* = .87, η_p_^2^ = 0.01], or predictability × probability [*F*(2.89, 75.17) = 1.38, *p* = .255, η_p_^2^ = 0.05].

**Table 3.** Mean reaction times (in ms), accuracy (%), and standard deviations (in parentheses) of the targets presented early, predicted, and late, separately for different standard intervals and probabilities.

Standard interval		Probability			Prediction effect		
		25%	50%	75%	25%	50%	75%
*Accuracy*							
Short	Early	97 (5)	97 (5)	99 (3)	2	1	0
	Predicted	99 (4)	98 (3)	99 (2)	–	–	–
	Late	98 (4)	98 (4)	98 (3)	1	0	1
Long	Early	98 (4)	97 (5)	99 (3)	1	1	0
	Predicted	99 (4)	98 (4)	99 (1)	–	–	–
	Late	98 (3)	99 (3)	99 (3)	1	−1	0
*Reaction times*							
Short	Early	456 (101)	486 (99)	521 (133)	54***	95***	143***
	Predicted	402 (114)	391 (108)	379 (122)	–	–	–
	Late	397 (118)	397 (105)	394 (111)	−5	6	15
Long	Early	409 (101)	417 (95)	426 (91)	36***	47***	55***
	Predicted	373 (94)	370 (95)	371 (94)	–	–	–
	Late	380 (100)	372 (104)	381 (96)	8	2	10

*Note.* Prediction effect was to subtract the predicted condition from the early and late conditions, respectively.

*p < .05, **p < .01, ***p < .001.

#### Reaction Times

Table 3 shows the RT versus the predictability in the short- and long-standard intervals for the different probabilities. There was a main effect of standard interval [*F*(1, 26) = 7.49, *p* = .011, η_p_^2^ = 0.22], with shorter RTs for long-interval trials (*M* = 388.69 ms, *SE* = 17.82) than for short-interval trials (*M* = 424.80 ms, *SE* = 19.97). There was also a main effect of predictability [*F*(1.22, 31.70) = 92.91, *p* < .001, η_p_^2^ = 0.78]. Further pairwise comparisons confirmed that the response was slowest when the target appeared in the early condition (*M* = 452.45 ms, *SE* = 17.37) compared to predicted (*M* = 380.94 ms, *SE* = 18.40, *p* < .001) or late unpredicted (*M* = 386.85 ms, *SE* = 18.37, *p* < .001) conditions, reflecting the temporal prediction effect.

There was no main effect of probability [*F*(1.47, 38.27) = 1.18, *p* = .306, η_p_^2^ = 0.04] or interaction effect of standard interval × probability [*F*(1.78, 46.38) = 0.34, *p* = .687, η_p_^2^ = 0.01]. However, there were standard interval × predictability [*F*(1.35, 35.01) = 16.76, *p* < .001, η_p_^2^ = 0.39] and probability × predictability interaction effects [*F*(2.97, 77.34) = 17.55, *p* < .001, η_p_^2^ = 0.40]. Interestingly, there was also a standard interval × probability × predictability interaction effect [*F*(2.57, 66.80) = 7.92, *p* = .001, η_p_^2^ = 0.23]. Further pairwise comparisons revealed that the RTs for early targets were longer than those for predicted targets (*p*s < .001) and late targets (*p*s < .003), regardless of the probability or standard interval. Thus, early-prediction effects were observed under all conditions, but no late-prediction effects were found.

A 2 (standard interval: short and long) × 3 (probability: 75%, 50%, and 25%) × 2 (prediction effect: early and late) repeated-measures ANOVA was conducted for prediction effects. The main effects of standard interval [*F*(1, 26) = 12.88, *p* = .001, η_p_^2^ = 0.33], probability [*F*(1.49, 38.83) = 18.91, *p* < .001, η_p_^2^ = 0.42], and prediction effect [*F*(1, 26) = 81.90, *p* < .001, η_p_^2^ = 0.76] were significant. And, the interaction effects of standard interval × probability [*F*(1.40, 36.40) = 7.42, *p* = .005, η_p_^2^ = 0.22], standard interval × prediction effect [*F*(1, 26) = 18.46, *p* < .001, η_p_^2^ = 0.42], probability × prediction effect [*F*(1.78, 46.22) = 16.60, *p* < .001, η_p_^2^ = 0.39], and standard interval × probability × prediction effect [*F*(1.47, 38.33) = 8.43, *p* = .002, η_p_^2^ = 0.25] were significant.

**Figure 3.**
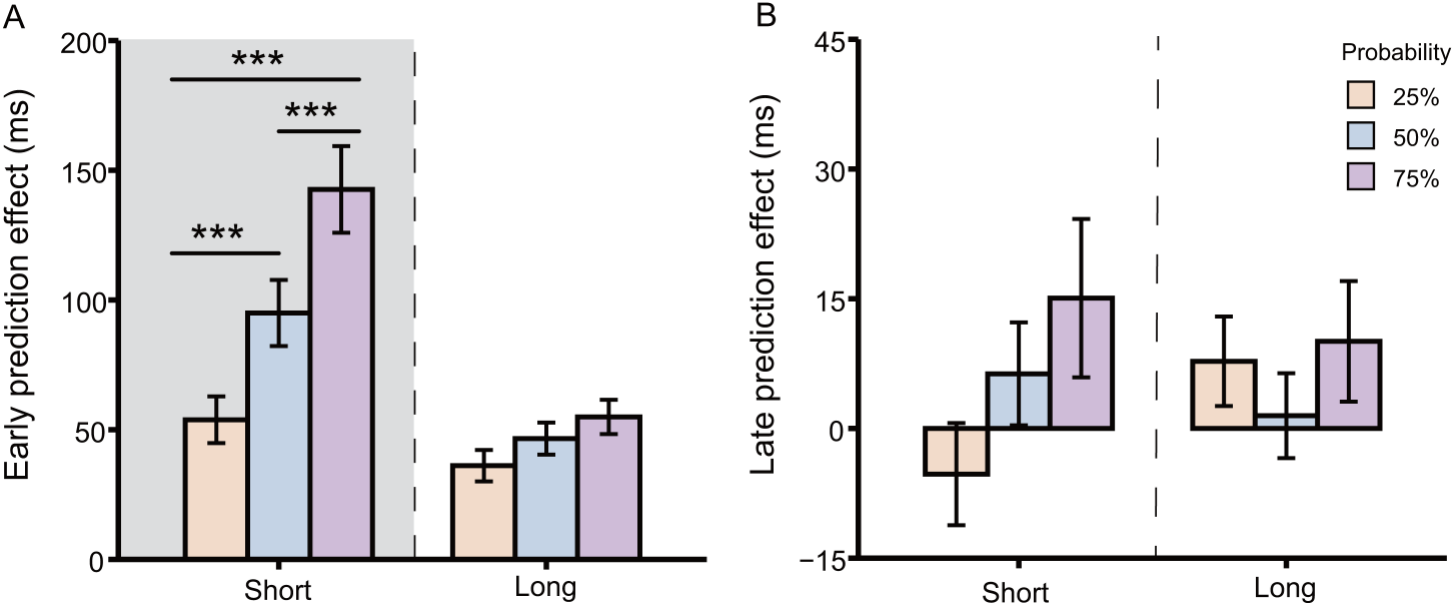
Prediction effect under all conditions. (A) Early-prediction effect for 75%, 50%, and 25% probabilities in the short- and long-standard interval conditions. (B) Late-prediction effect for 75%, 50%, and 25% probabilities in the short- and long-standard interval conditions. Error bars represent standard errors of the means.

*Note.* *p < .05, **p < .01, ***p < .001.

For early-prediction effect, 2 (standard interval: short and long) × 3 (probability: 75%, 50%, and 25%) repeated-measures ANOVA was conducted (Figure 3A). A main effect of the standard interval [*F*(1, 26) = 18.85, *p* < .001, η_p_^2^ = 0.42] was found, with greater early-prediction effect for short-interval trials (*M* = 97.14 ms, *SE* = 11.44) than for long-interval trials (*M* = 45.87 ms, *SE* = 4.70). There was also a main effect of probability [*F*(1.82, 47.36) = 33.94, *p* < .001, η_p_^2^ = 0.57]. Importantly, a significant interaction effect between the standard interval and probability was found [*F*(1.40, 36.43) = 12.75, *p* < .001, η_p_^2^ = 0.33]. In the short-interval condition, the early-prediction effect was stronger in the 75% probability condition (*M* = 142.62 ms, *SE* = 16.71) than in the 50% probability condition (*M* = 94.99 ms, *SE* = 12.75, *t*_(26)_ = 4.36, *p* < .001, *d* = 0.73) and 25% probability condition (*M* = 53.82 ms, *SE* = 8.99, *t*_(26)_ = 6.34, *p* < .001, *d* = 1.37), and the early-prediction effect was stronger in the 50% probability condition than in the 25% probability condition (*t*_(26)_ = 4.81, *p* < .001, *d* = 0.63). However, in the long-interval condition, there was no significant difference in the early-prediction effect among probabilities (*p*s > .068). In summary, early-prediction effects were modulated by probability only in the short-interval condition and not in the long-interval condition, and the early-prediction effect was strongest in the 75% probability condition, followed by that in the 50% probability condition, with the weakest prediction effect observed in the 25% probability condition.

For late-prediction effect, 2 (standard interval: short and long) × 3 (probability: 75%, 50%, and 25%) repeated-measures ANOVA was conducted (Figure 3B). No main effects of the standard interval [*F*(1, 26) = 0.04, *p* = .85, η_p_^2^ = 0.001], or probability [*F*(1.37, 35.52) = 1.72, *p* = .20, η_p_^2^ = 0.06] were found. And no interaction between standard interval and probability was found [*F*(1.45, 37.58) = 1.38, *p* = .259, η_p_^2^ = 0.05]. That is, late-prediction effect was not moderated by probability regardless of short or long intervals.

In summary, the results of Experiment 2 found that the early-prediction effect was found regardless of interval and probability. And the early-prediction effect was moderated by probability under the short-interval condition, with the effect size increasing as probability increased. However, no late-prediction effects were observed in any condition.

## Discussion

Two experiments were conducted to determine how temporal predictions are influenced by conditional probabilities in tactile perceptual processing. Experiment 1 replicated earlier findings, demonstrating that temporal predictions can be generated with a constant foreperiod paradigm. We also found that prediction effects occurred for foreperiods of 500 and 1,000 ms. In Experiment 2, we used 500 and 1,000 ms as short and long intervals, respectively. Overall, the results of this study show that temporal predictions and conditional probabilities do interact, but only when the interval between the cue and target stimuli is short. When the interval between the cue and target stimuli is long, temporal predictions and conditional probabilities are independent.

The first objective was to explore the effects of temporal predictions on tactile perception. This experiment was performed by using a rhythmic temporal sequence; that is, the duration of the cue-target interval (foreperiod) was fixed. In Experiment 1, the duration of the foreperiod was either fixed (predicted condition) or varied (unpredicted condition). The RT analyses showed better performance for predicted foreperiods than for unpredicted foreperiods, confirming that temporal predictions can be generated with a constant foreperiod, which is consistent with the results of previous studies (Koppe et al., 2014; Niemi & Näätänen, 1981). In addition, this was also consistent with false alarm rate results. Participants were more likely to react to catch trials in predicted condition than unpredicted condition. The common explanation for this phenomenon is that predictable foreperiods lead to increased preparation time, and, thus greater temporal certainty (Capizzi et al., 2015). In the constant foreperiod paradigm, a single foreperiod is used within a trial block. Participants can predict the occurrence of time points well and prepare for a reaction accordingly. In the variable foreperiod paradigm, foreperiods vary among trials within a block, with uniform uncertainty for all foreperiods. Therefore, participants could not accurately predict when the target stimulus would appear and thus could not prepare for a response.

The temporal prediction effect occurs only when the foreperiods are 500 and 1,000 ms and gradually weakens as the foreperiod duration increases. According to a previous study, temporal prediction effects are typically much greater for (or restricted to) targets that appear after short foreperiods (Anderson & Sheinberg, 2008; Correa & Nobre, 2008; van Ede et al., 2020). The most parsimonious explanation for this phenomenon is that as the time following a warning signal increases, uncertainty decreases, leading to shorter RTs during longer foreperiods. In addition, no temporal prediction effect was observed at long foreperiods, and there is a possibility that the experiment is not sufficiently powered to observe a statistically significant effect in 1,500 and 2,000 ms.

In Experiment 2, participants were presented with a rhythmic sequence before the blocks, and targets were presented either in- or out-of-synchrony (earlier or later) with this rhythmic sequence in the blocks. Tactile targets were detected rapidly when their onset coincided with moments previously practiced by the rhythmic sequences in both the short- and long-interval conditions. This result suggests that temporal predictions can be flexibly tuned to different intervals on the basis of the previously established rhythm (Correa & Nobre, 2008). The finding explains the absence of a foreperiod effect with the fastest RTs for late targets and the slowest RTs for early targets in both the short- and long-interval conditions, which is inconsistent with previous studies (Van Ede et al., 2011; Xu et al., 2021). In our study, the RTs for predicted and late targets were equally fast. There are several possible explanations for the observed results.

One reason may be how the rhythmic information was presented. In previous studies, rhythmic information was presented in a trial-by-trial manner. In each trial, participants reconsolidated rhythmic information. However, in the present study, the rhythmic information was presented only before blocks, and the participants could rely only on the preexisting rhythmic information to respond, resulting in the weakening of the rhythmic information. This results in no difference between the rhythmic (predicted) and postrhythmic (late unpredicted) results. Another reason may be the hazard function effect. As time passes, the probability of a target appearing later than expected increases, giving participants more time to prepare and leading to faster RTs for late targets. The third possibility is the generalization effect of temporal predictions (Thomaschke et al., 2011b). The temporal anticipation of the rhythm is spread backward to the time points surrounding the rhythm regardless of long and short foreperiods. Although there are a variety of possible explanations for the lack of RT effects when targets are presented at the predicted or later time points, which are not mutually exclusive, further research is necessary to confirm any of these effects.

The second objective was to investigate how temporal predictions interact with conditional probabilities in tactile perceptual processing. In Experiment 2, the modulation of the tactile temporal prediction effect by the probability was restricted to the case in which the foreperiod was short, and the target appeared earlier than the predicted moment. This shows that uninformative cues can be used to guide tactile predictions, similar to what has previously been demonstrated using a single symbolic tactile cue (Jones & Forster, 2013, 2014). Furthermore, participants can learn temporal regularities between cues and targets. When the target is presented earlier than the predicted moment, the participants are easily aware that the learned time regularity has been violated, resulting in a slower RT.

The present behavioral analysis showed that the difference in RTs (early vs. predicted conditions) increased as the proportion of predicted trials per block increased in the short-interval condition. This result is in line with previous studies using visual stimuli (Arjona et al., 2016; Vossel et al., 2006), and now the tactile modality can be added to the list. This behavioral pattern could be explained by an increase in the sensory-motor preparation period as the percentage of predicted moment trials per block increases. That is, the distribution of processing resources in anticipation of a highly predicted moment would have a greater peak at the predicted moment and a lower value at the early moment than in the condition with an unpredicted moment. This would make the transfer of processing resources more difficult and hence increase the prediction effect for the highly predicted ratio. Moreover, no effect of probability on the temporal prediction was observed at the *p* < .05 level in the long-interval condition. We cannot exclude a possible difference in early-prediction effects between a 75% probability and a 25% probability (*p* = .068) due to lower statistical power. In the following research, we will further explore this issue.

In conclusion, this study revealed the effects of temporal prediction induced by rhythmic stimuli on tactile perception and the influence of conditional probability. First, we explored the range of foreperiods in which temporal prediction effects can be found. This prediction effect was observed only when the foreperiods were 500 and 1,000 ms. Next, we investigated the benefits of temporal prediction on tactile perception while manipulating the conditional probability (setting the stimulus onsets earlier than, at, or later than the predicted moment in short and long intervals). Participants showed significantly greater prediction effects in the high-predicted probability condition than in the low-predicted probability condition when foreperiod was short for early targets, indicating slower transfer of processing resources in the high-predicted probability condition.
